# Opioid Dependent and Pregnant: What Are the Best Options for Mothers and Neonates?

**DOI:** 10.1155/2012/195954

**Published:** 2012-02-07

**Authors:** Annemarie Unger, Verena Metz, Gabriele Fischer

**Affiliations:** Department of Psychiatry and Psychotherapy, University Hospital of Vienna, 1090 Vienna, Austria

## Abstract

Pregnancy in opioid-dependent women is a major public health issue. Women who are afflicted by opioid addiction are a highly vulnerable group of patients frequently becoming pregnant unplanned and at risk of adverse pregnancy outcomes and peri-natal complications. Opioid agonist maintenance treatment is the best option for the majority of women. Ideally, early and closely monitored treatment in an interdisciplinary team approach including social workers, nurses, psychologists, psychiatrists, gynecologists, anesthesiologists, and pediatricians should be provided. The treatment of comorbid psychiatric conditions, the resolution of financial, legal, and housing issues, and the psychosocial support provided have a significant effect on optimizing pregnancy outcomes. This paper aims to update health professionals in the field of gynecology and obstetrics on the latest optimal treatment approaches for mothers suffering from opioid dependence and their neonates.

## 1. Introduction

Illicit drug use among pregnant women is an international health issue that has become of increasing relevance in the past decades. In 2001, a national household survey reported 3.7% of pregnant women in the United States using illicit drugs [1]. In 2009 this number had increased to 4.5% [2]. The actual numbers are higher if estimated numbers of unreported cases are taken into account due to fear of stigmatism impairing self-report measures. In fact, a study of maternal urine samples at delivery of 715 women in Florida showed 13.3% were positive for an illicit drug such as marijuana, cocaine, or opiates. Although among illicit substances, the prevalence of substance abuse among pregnant women was highest for marijuana, followed by cocaine [3], the prevalence of opioid dependence was on the rise between 1970 and 1980 [4] and an estimated 8 million people worldwide were reported to abuse opioids in 2003 [5]. Though men still outnumber women, the proportion of women continues to increase, and more than 70% of opioid-dependent women are of child-bearing age. Unplanned pregnancies are common due to effects of opioids on the female reproductive system, frequently leading to irregular menstruation or amenorrhea [6]. Additionally, chaotic lifestyles associated with drug abuse often foster insufficient birth control measures and consequently unexpected pregnancies. In fact, the rate of unintended pregnancies has been found to range between 80% and 90% among opioid-dependent women [7].

## 2. Opioid Dependence and Pregnancy

Opioid-dependent women commonly face numerous socioeconomic problems such as unemployment, coaddicted partners, and partner violence [8]. Prostitution as a means of attaining drugs often leads to health problems such as infectious disease or these occur as a consequence of syringe sharing and are poorly attended to [9]. The majority of opioid-dependent women suffer cooccurring psychiatric disorders with prevalence numbers ranging between 56 and 73%, mainly affective disorders, PTSD, or personality disorders [9–11] (see Table 1).

**Figure 1:**
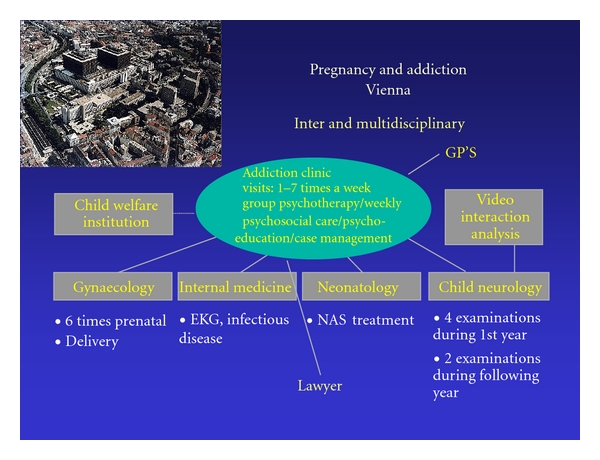
The multiprofessional team approach at the Addiction Clinic, University hospital of Vienna, Department of Psychiatry.

The failure to recognize mental disorders is a major risk to the health of mother and neonate as comorbid depression, anxiety disorders, and psychosis are associated with a variety of negative pregnancy outcomes [13]. Examples of these are preterm labor and poor fetal growth [14, 15], a heightened risk for perinatal complications and dysfunctional mother-child bonding [16]. Ideally, comorbid psychiatric conditions should be adequately treated, and the use of antidepressant drugs such as SSRI medication, which has been shown to be safe during pregnancy, may be indicated. However, a careful risk-benefit evaluation of pharmacological treatment by a psychiatric professional experienced in treating pregnant women is warranted [17]. It should be part of a multiprofessional team approach comprising psychiatrists, psychologists, gynecologists, midwifes, nurses, social workers, and anesthesiologists. (see Figure 1). 

## 3. Medical Treatment of Opioid-Dependent Pregnant Women

Though ultimately, abstinence from opioids might seem the best option during pregnancy, few opioid-addicted women can handle abstaining from opioids at such a time in their lives filled with changes and stress. Additionally, rapid detoxification during pregnancy cannot be recommended from a medical standpoint, as withdrawal has been linked to intrauterine stress for the fetus associated with poor fetal growth, preterm delivery, and fetal death [18]. Though gradual detoxification in the second or third trimesters has been achieved in a selected group of women, the majority of women have a high risk of relapse. The best option for most opioid-addicted pregnant women is opioid maintenance treatment with a long-acting synthetic opioid such as methadone or buprenorphine [19–24]. Methadone has for a long time been the established maintenance medication for pregnancy [25, 26], however, in recent years, buprenorphine has been increasingly subject of studies as a valuable alternative to methadone with beneficial effects on the neonatal abstinence syndrome of the newborn. 

Pioneer work with standardized prospective evaluation on the use of buprenorphine in pregnant women has been conducted in the late 1990s by Fischer et al. at the Addiction Clinic in Vienna, who was first to publish a study demonstrating maternal and fetal safety of women maintained on buprenorphine during pregnancy and consecutively during conception [22, 27]. The pilot study published in 2000 was followed by a double-blind, double dummy comparison study of 14 women published in 2006, forming a first basis for larger follow-up studies and showing higher retention rates in the buprenorphine group. So far, the largest double-blind, double dummy study comparing the safety and efficacy of buprenorphine versus methadone in pregnant opioid-dependent women was the “MOTHER study” (Maternal Opioid Treatment: Human Experimental Research). It was conducted between 2005 and 2008 as a multisite randomized controlled trial encompassing 6 US American sites and one European site at the Addiction Clinic, University Hospital of Vienna, Austria. The main outcome of this trial of 131 completers and their neonates was recently published in the New England Journal of Medicine [28], finding that neonates prenatally exposed to buprenorphine had a significantly shorter duration of treatment and required significantly lower amounts of morphine medication compared to methadone-exposed neonates. The shorter duration of hospitalization of buprenorphine-exposed neonates should also be seen in the light of health service costs, as large numbers of neonates are affected by NAS every year. However, treatment with methadone still has its place as 28 of 86 women in the buprenorphine group (33%) discontinued treatment compared to 16 of 89 women (18%) maintained on methadone [28]. Buprenorphine can be seen as an important treatment option for this target group, but methadone continues to be the medication for those women who do not positively respond to buprenorphine. 

Another important aspect affecting pregnant opioid-maintained women that deserves mention is the need for dose adjustment in the majority of women, usually around the beginning of the third trimester, due to changes in metabolic rates, increased estrogen levels, and enzyme induction [29, 30]. After delivery, most women will request dose decreases due to changes in hormonal status. The same principle applies to the use of psychotropic medications during pregnancy, such as SSRIs, where frequently dose adjustment is necessary, and psychiatric symptoms can worsen temporarily.

Recent reports have shown that not only the dose but also the duration of maintenance treatment during pregnancy plays a role in improvement of pregnancy outcomes, as in a recent study women who had been on methadone maintenance treatment (MMT) for the full pregnancy had higher birth weights and a higher likelihood of abstinence from concomitant medication associated with higher gestational age at delivery compared to women who had only been on MMT for part of their pregnancy [31]. Results from studies like this emphasize the responsibility of gynecologists to seek close cooperation with addiction specialists as soon as pregnancy is ascertained. 

## 4. Perinatal Pain Management of Opioid-Dependent Women

Opioid dependence is associated with heightened sensitivity to pain, chronic hyperalgesia, and tolerance to opioid pain medication [32], making peripartum pain management of opioid-dependent women particularly challenging. There is a persistent lack of standardized treatment recommendations, stigma, and overcaution due to fear of “drug-seeking” behavior, resulting in undertreatment of peripartum pain in the majority of cases. Additional factors such as nicotine addiction also contribute to heightened pain sensitivity [33, 34]. This is significant given that more than 90% of opioid-dependent women are smokers [35]. Due to abrupt nicotine deprivation in the hospital, they often require higher doses of pain medication [36]. Furthermore, the high prevalence of other psychiatric diagnoses, such as affective disorders, represent an additional independent predictor of intensified pain experience [32, 37]. Prior recommendations on pain treatment of opioid-dependent patients have stressed the importance of continuous, adequately dosed maintenance treatment as a basis [38]. Recent findings support this recommendation and, additionally, the use of NSAIDs (NonSteroidal Anti-Inflammatory Drugs) to supplement, and the use of opioids, other than the ones used for maintenance treatment, for sufficient pain control during delivery and postpartum [32, 39]. The use of NSAIDs cannot be recommended during pregnancy, particularly during the third trimester due to the risk of early closure of the ductus arteriosus. In early pregnancy the use of NSAIDs has been linked to increased risk of miscarriage and premature birth [40]. 

## 5. The Neonatal Abstinence Syndrome of the Newborn 

The neonatal abstinence syndrome of the newborn is a condition which becomes manifest in the first few days after delivery affecting more than half of newborns born to opioid-dependent mothers [41]. It is characterized by symptoms affecting primarily the central nervous system, the respiratory system, and the digestive tract. The first scale for measurement of neonatal abstinence syndrome was developed by Loretta Finnegan et al. in the early 1970s [42]. It consists of symptoms such as increased sneezing, watery eyes, frequent yawning, poor sucking, reduced sleep duration after feeding, and increased (hyper) reflexes. Management of this condition is best handled in a multiprofessional team approach in specially trained centers, where symptoms of neonates are rated at regular intervals and, if needed, treatment is initiated using morphine hydrochloride drops given to infants. In 2007, Fischer and colleagues at the Vienna addiction clinic published a study comparing phenobarbital to morphine hydrochloride as NAS treatment medication, showing that the majority of neonates had a significantly shorter duration of NAS under morphine hydrochloride drops which has become the established form of treatment [43]. However, prolonged use of morphine in the postnatal period should be avoided as recent studies have demonstrated a number of negative consequences of opioids on neural cells of the growing brain [44]. 

A recently updated Cochrane review by Osborn referring to treatment options in 645 infants confirmed that opiate (morphine) treatment is superior to supportive care only. The analysis of prior studies also showed that opiate treatment was also superior to phenobarbitone and to diazepam in terms of rates of treatment failure and in reducing the likelihood of seizures [41]. The basis for effective medical treatment of NAS is the standardized rating of symptoms in neonates and supportive nonpharmacological interventions [45].

## 6. Individual Predisposition to NAS 

A question which has not been resolved despite numerous hypotheses is why some neonates will develop symptoms of neonatal abstinence syndrome, and others will not. The majority of studies that have examined the association between maternal maintenance dose and NAS have found no association [21, 23, 27, 46–50]. A most recent review and meta-analysis of 67 studies on maternal methadone dose and NAS, and another encompassing 10 studies with various medications, support prior findings, also not reporting any correlation [51, 52]. Factors that do have an impact on NAS and neonatal outcomes are concomitant consumption of opioids, cocaine, or other substances. In particular, benzodiazepine consumption has been associated with prolonged neonatal abstinence syndromes [53]. Another factor, which has been shown to play an important role during pregnancy, is the use of nicotine, as over 90% of opioid-dependent pregnant women are strongly dependent on nicotine [35, 54]. The prescription use of SSRIs and other psychotropic medications also has a negative impact on NAS [55]. The role of neonatal gender in NAS occurrence has been addressed in two prior studies which found inconclusive results. One study of a population of 64 neonates exposed to methadone found that male neonates exhibited a higher NAS intensity in the first four days postpartum, however, no sex-specific differences in rates of NAS treatment were found [55]. The second study reported no significant sex-related differences in a retrospective chart review of 308 methadone-exposed neonates [56]. 

Though some factors have been determined that can be seen as predictors of NAS, a lot of questions around this topic remain unresolved. There is a good chance that answers may actually rather be found on a more complex level such as biochemical processes in the placenta. Genetic variations of certain placental transporter genes could explain levels of maternal opioids in the fetal circulation during pregnancy, which in turn could explain severity and incidence of NAS [57, 58]. However, further research is needed to clarify these findings, and it is also questionable how such genetic findings can contribute to therapeutic options. 

## 7. Breastfeeding and Maintenance Treatment

Breastfeeding under oral opioid agonist treatment is recommendable for women if they are not comorbidly suffering from active forms of infectious disease such as hepatitis C with high blood viral loads; HIV is a definite contraindication. For methadone, breastfeeding can reduce the severity and duration of NAS and delay the onset of symptoms [59–61]. One reason for this can be the comfort obtained through mother/child bonding, another, the oral bioavailability of methadone. As a result of breast feeding under methadone maintenance, the need for medical treatment of NAS may be decreased [61]. If women want to breastfeed and no contraindication such as either infectious disease or continued illicit drug use exists, physicians should support their needs [62–64]. The safety of buprenorphine in breastfeeding has not been well-investigated. So far, data available show low concentrations in breast milk [65] so that a similar recommendation might be valid as for methadone. Nonetheless, though findings so far are supportive, they are restricted by shorter periods of observation and smaller numbers of investigated cases [66].

## 8. Conclusion

Opioid-dependent pregnant women are a highly vulnerable group of patients who frequently have unplanned pregnancy [7] and are at risk of unfavorable outcome and peri-natal complications. They often suffer comorbid psychiatric disorders that need to be recognized and treated adequately in order to avoid complications during pregnancy and ensure a chance for healthy mother-child interaction. The majority of women benefits most from opioid agonist maintenance therapy with a long-acting synthetic opioid such as methadone or buprenorphine. The management of pain during delivery and thereafter is often challenging due to opioid tolerance and heightened pain sensitivity. Opioid-dependent pregnant women are in need of early and closely monitored treatment in a multiprofessional team approach including social workers, nurses, psychologists, psychiatrists, gynecologists, anesthesiologists, and pediatricians. The resolution of financial, legal, and housing issues, and the psychosocial support provided through treatment have a significant effect on optimizing pregnancy outcomes. 

## Figures and Tables

**Table 1:** Psychiatric* comorbidity* in substance abuse treatment and matched controls*.

	SA	Controls
Depression	36.3%	4.2%
Anxiety disorder	16.3%	2.3%
ADHD	17.2%	3.0%
Conduct disorder	19.3%	1.2%
Conduct disorder (w/ODD)	27.3%	2.3%
Any psychiatric diagnosis	55.5%	9.0%

* All *P* < .001 [12].
